# Myeloid Takl Acts as a Negative Regulator of the LPS Response and Mediates Resistance to Endotoxemia

**DOI:** 10.1371/journal.pone.0031550

**Published:** 2012-02-14

**Authors:** Christina Eftychi, Niki Karagianni, Maria Alexiou, Maria Apostolaki, George Kollias

**Affiliations:** Institute of Immunology, Biomedical Sciences Research Center “Alexander Fleming”, Vari, Greece; French National Centre for Scientific Research, France

## Abstract

TGFβ-activated kinase 1 (TAK1), a member of the mitogen-activated protein kinase kinase kinase (MAP3K) family, is considered a key intermediate in a multitude of innate immune signaling pathways. Yet, the specific role of TAK1 in the myeloid compartment during inflammatory challenges has not been revealed. To address this question, we generated myeloid-specific kinase-dead TAK1 mutant mice. TAK1 deficiency in macrophages results in impaired NF-κB and JNK activation upon stimulation with lipopolysaccharide (LPS). Moreover, TAK1-deficient macrophages and neutrophils show an enhanced inflammatory cytokine profile in response to LPS stimulation. Myeloid-specific TAK1 deficiency in mice leads to increased levels of circulating IL-1β, TNF and reduced IL-10 after LPS challenge and sensitizes them to LPS-induced endotoxemia. These results highlight an antiinflammatory role for myeloid TAK1, which is essential for balanced innate immune responses and host survival during endotoxemia.

## Introduction

TGFβ-activated kinase 1 (TAK1) was originally identified as a key regulator of TGFβ/bone morphogenetic protein signaling [1]. Since then a wealth of information has been generated placing TAK1 as part of a more complex signaling network that governs basic cellular activities. Studies using complete and conditional TAK1 knockout mice revealed that TAK1 integrates signals emanating from TGFβ, TNF, IL-1β, Toll-like receptors (TLRs), B-cell receptors and T-cell receptors, to coordinate homeostasis and immunity, and that its absence can lead to carcinogenesis, inflammation or death [2]–[13]. In Drosophila, TAK1 is critical for antibacterial innate immunity as TAK1 mutants are highly susceptible to Gram-negative bacterial infection and do not produce antibacterial peptides [14]. In mammals, there are several lines of evidence supporting a critical role for TAK1 in innate immunity [8], [11], [15]. Genetic evidence demonstrating a role for TAK1 during innate immunoreceptor signaling has been obtained in mature immune cells by using B cell-specific TAK1-deficient mice [8]. TAK1-deficient B cells fail to activate transcription factor NF-κB and mitogen-activated protein kinases (MAPKs) in response to TLR ligands and have impaired production of IL-6, supporting an evolutionary conserved role for TAK1 in innate immunity. However, the role of TAK1 during TLR innate immune responses has not been addressed in the main cellular mediators of innate immunity, the myeloid cells.

Myeloid cells (macrophages and neutrophils) are the chief cellular agents of the inflammatory cascade during microbial infection. They initiate coordinated innate immune defenses through activation of pathogen recognition receptors that recognize specific pathogen-associated molecular patterns [16]. As a result of these interactions, immune cellular activation occurs with the release of cytokine and non-cytokine mediators. A key event in the immune response to Gram-negative bacteria is the recognition of lipopolysaccharide (LPS) by TLR4 [17]. LPS plays a key role in Gram-negative sepsis by inducing production of proinflammatory and antiinflammatory mediators, the most critical being IL-1β, TNF, IL-6 and IL-10 [18]. Cytokine production significantly influences the quality, duration, and magnitude of most inflammatory reactions.

During LPS-induced endotoxemia, serine/threonine kinase cascades are activated with pleiotropic downstream effects that include activation of protein kinases such as the MAPKs and the I-κB kinases. Although key molecules in these signaling pathways have been identified, there are still substantial gaps in our knowledge, including the role of members of the MAPK kinase kinase (MAP3K) family. Here we investigated the myeloid-specific role of the MAP3K TAK1 during LPS inflammatory responses. TAK1 deficiency in macrophages led to impaired activation of NF-κB and JNK following LPS stimulation, identifying TAK1 as an important upstream signaling molecule that regulates LPS-induced NF-κB and JNK activation in macrophages. Cytokine profile analysis of TAK1-deficient macrophages upon stimulation with LPS, revealed a hyperinflammatory phenotype characterized by increased proinflammatory (IL-1β, TNF, and IL-6) and reduced antiinflammatory (IL-10) cytokine production. A similar inflammatory cytokine profile was observed in LPS-stimulated neutrophils, although no reduction in IL-10 production was observed. Consistent with the above, mice with defective myeloid TAK1 mount an enhanced innate immune response to LPS by exhibiting increased circulating levels of IL-1β and TNF, reduced IL-10, and subsequently significantly increased mortality to LPS-induced shock. We conclude that myeloid TAK1 acts by regulating the balance between proinflammatory and antiinflammatory cytokine production thereby preventing unrestrained inflammatory responses.

## Results and Discussion

### Generation of ubiquitous and myeloid-specific TAK1 mutant mice

**Figure 1:**
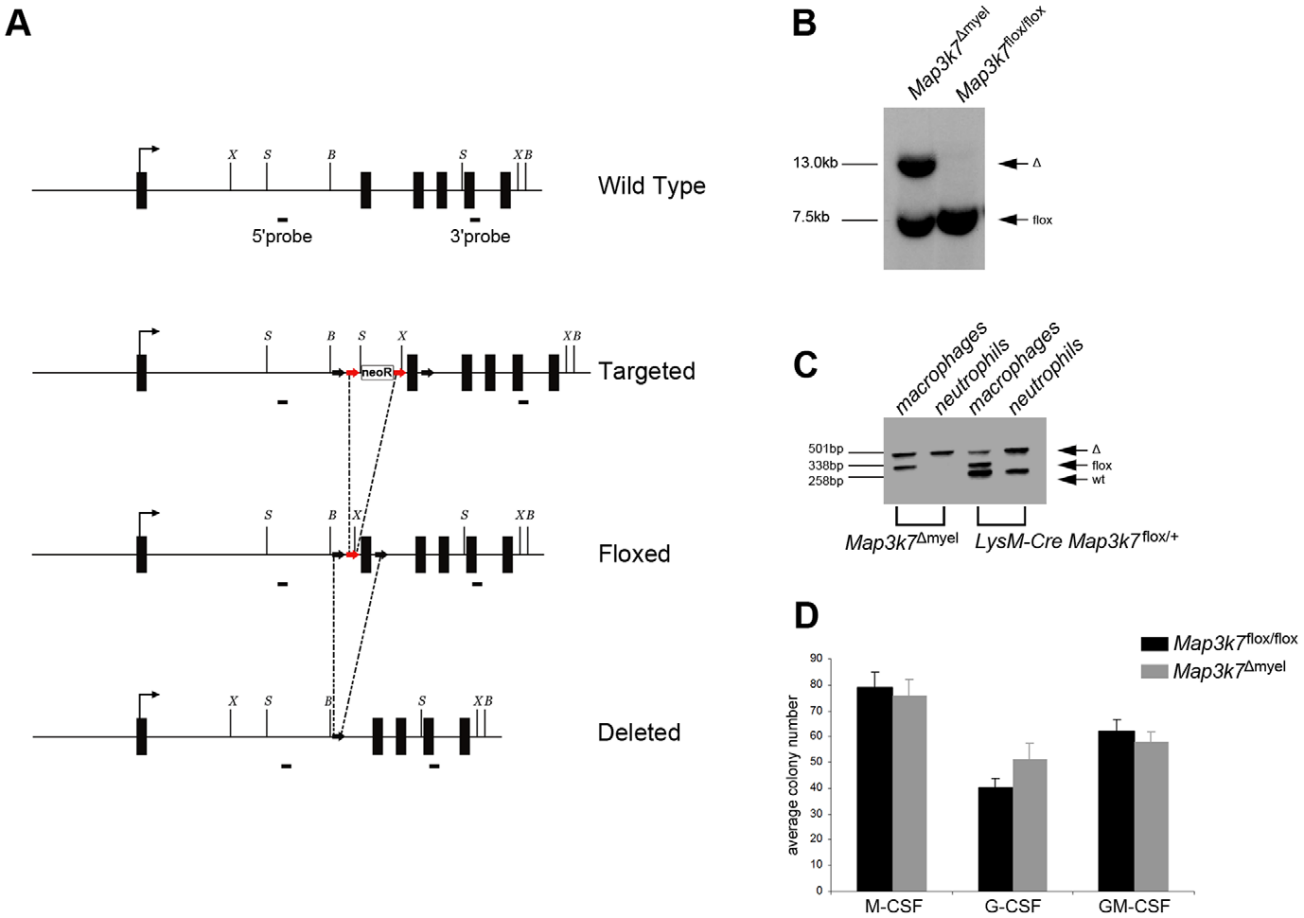
Generation of conditional TAK1-deficient mice. (A) Schematic representation of the wild type, targeted, floxed and deleted *Map3k7* genomic locus, indicating BamHI, SacI and XbaI restriction sites used for Southern blot. The *Map3k7* locus comprises 17 exons; in the scheme the first 6 exons are represented. The probes used to verify homologous recombination at the 5′ and 3′ end are shown. Black arrows indicate loxP sites; red arrows indicate FRT sites. B, BamHI; S, SacI; X, XbaI. (B) Southern blot of genomic DNA isolated from BMDMs after digestion with XbaI and using the 3′ probe. (C) PCR analysis of genomic DNA isolated from FACS-sorted CD11b^+^F4/80^+^ resident peritoneal macrophages and CD11b^+^Gr1^+^ splenic neutrophils. (D) Colony formation by BM cells from *Map3k7* ^Δmyel^ and *Map3k7* ^flox/flox^ mice in response to M-CSF, G-CSF and GM-CSF. Data are shown as mean ± SEM of 4 mice per group and are representative of two independent experiments.

We generated mice with conditional expression of a *Map3k7* allele encoding a kinase-dead truncated form of TAK1, following a similar targeting strategy to previous studies [8]. The targeting vector was constructed by placing two loxP sites flanking exon 2 of *Map3k7* (Fig. 1A). Exon 2 encodes part of the kinase domain including the ATP binding pocket and can be deleted without disrupting the remainder of the reading frame. To generate mice that ubiquitously express the truncated TAK1 (*Map3k7* ^−/−^ mice), we crossed *Map3k7* ^flox/flox^ mice with transgenic mice expressing Cre in germ cells [19]. Of 101 newborn pups (19 litters) obtained by intercrossing *Map3k7* ^+/−^ mice, we had 71 *Map3k7^+^* ^/−^, 30 *Map3k7^+/+^* and no *Map3k7* ^−/−^ mice, confirming previous studies that TAK1 deficiency leads to embryonic lethality [4], [8], [11].

To investigate the role of myeloid-specific TAK1 in innate immunity, we crossed *Map3k7* ^flox/flox^ mice with *LysM-*Cre knockin mice, which express the Cre recombinase in macrophages and neutrophilic granulocytes [20]. This resulted in a 50% deletion in macrophages as it was assessed by Southern blot analysis of DNA extracted from bone marrow-derived macrophages (BMDMs) (Fig. 1B). Additionally, PCR analysis was performed on FACS-sorted CD11b^+^F4/80^+^ resident peritoneal macrophages and CD11b^+^Gr1^+^ splenic neutrophils for an approximate indication of deletion, suggesting a similar percentage of deletion in the macrophage population, while a higher deletion percentage was observable in neutrophils (Fig. 1C). Mice with myeloid-restricted TAK1 deficiency (*Map3k7* ^Δmyel^) were born at the expected Mendelian ratio, developed normally, and did not show any gross morphological changes in the overall histology of lymphoid organs and other organs rich in myeloid cells, such as the gastrointestinal tract (data not shown). In the control groups, we observed no phenotypic difference among *Map3k7* ^flox/flox^ and *LysM-*Cre mice and thus *Map3k7* ^flox/flox^ mice were used as controls.

To determine if TAK1 deficiency affected the production of immature and mature myeloid cell subsets, we performed flow cytometric immunophenotypical analysis of bone marrow (BM) [21], [22], peripheral blood, spleen and peritoneal cavity cell preparations obtained from *Map3k7* ^Δmyel^ and *Map3k7* ^flox/flox^ mice. We observed no differences in the immature myeloid cell subsets in the BM of *Map3k7* ^Δmyel^ and *Map3k7* ^flox/flox^ mice as there were similar frequencies of common myeloid progenitors, granulocyte/macrophage progenitors and the myelomonocytic cell fraction (fraction of Gr-1^+^/Mac-1^+^ cells) (Table 1). In peripheral blood there were similar numbers of monocytes and neutrophils between *Map3k7* ^Δmyel^ and *Map3k7* ^flox/flox^ cell preparations (Table 1). Also, no statistically significant differences were observed in the number of peripheral splenic macrophages and neutrophils, nor in the number of resident peritoneal macrophages (Table 1).

**Table 1:** Immunophenotypical flow cytometric analysis of BM, peripheral blood, spleen and peritoneal cavity myeloid cells in *Map3k7* ^Δmyel^ and *Map3k7* ^flox/flox^ mice.

	*Map3k7* ^flox/flox^ (n = 7–8)	*Map3k7* ^Δmyel^ (n = 7)
**BM (×10^9^)**		
CMPs (Lin^−^c-Kit^+^Sca-1 CD16/32^lo^CD34^+^)	1.4±0.4	1.3±0.3
GMPs (Lin^−^c-Kit^+^Sca-1^−^CD16/32^hi^CD34^+^)	2.4±0.5	2.2±0.3
Gran/Mac fraction (Gr1^+^/CD11b^+^)	1.5±0.3	1.5±0.1
**Peripheral Blood (%)**		
Monocytes (CD11b^+^Ly6C^hi^)	0.9±0.3	1.4±0.6
Neutrophils (CD11b^+^Ly6C^lo^Gr1^hi^)	3.3±0.7	2.7±1.5
**Spleen (×10^6^)**		
Macrophages (F4/80^+^)	134±49	137±78
Neutrophils (CD11b^+^Gr1^+^)	100±45	186±144
**Peritoneal cavity (×10^6^)**		
Resident macrophages (CD11b^+^F4/80^+^)	56±17	60±19

Mean values ± SD obtained from the indicated number of mice (n) are given. Measurements are a pool of two independent experiments. CMPs, common myeloid progenitors; GMPs, granulocyte/macrophage progenitors.

We next investigated the ability of myeloid precursor cells to differentiate into macrophages and granulocytes in colony-forming assays upon stimulation with G-CSF, M-CSF, and GM-CSF. As shown in Fig. 1D, the numbers of colonies grown from *Map3k7* ^Δmyel^ and *Map3k7* ^flox/flox^ BM cells were comparable, suggesting that myeloid TAK1 deficiency did not affect the developmental potential of early myeloid precursor cells. It has been reported that TAK1 promotes survival signals in hematopoietic cells [12]. Nevertheless, we and others [15] did not observe any reduction in the numbers of macrophages and granulocytes in the periphery when TAK1 is inactivated in myeloid cells.

### TAK1 deficiency promotes a hyperinflammatory phenotype in LPS-stimulated macrophages and neutrophils

**Figure 2:**
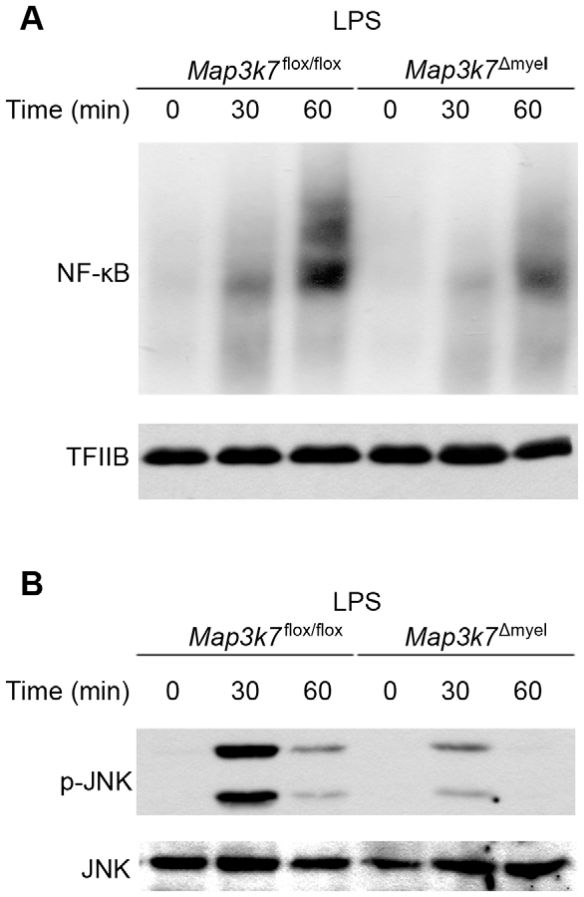
Impaired activation of NF-κB and JNK in response to LPS stimulation in TAK1-deficient macrophages. BMDMs were stimulated with LPS (100 ng/ml) and nuclear/cytoplasmic extracts were collected at the indicated times. (A) NF-κB DNA-binding activity in nuclear extracts was determined by EMSA. The lysates used for EMSA were subjected to immunoblot analysis using a TFIIB-specific antibody as a loading control. (B) Phosphorylation of JNK (p-JNK) in cytoplasmic extracts was assessed by immunoblot with antibody specific for its phosphorylated form. The membrane was reprobed for total JNK. Results are representative of three independent experiments.

**Figure 3:**
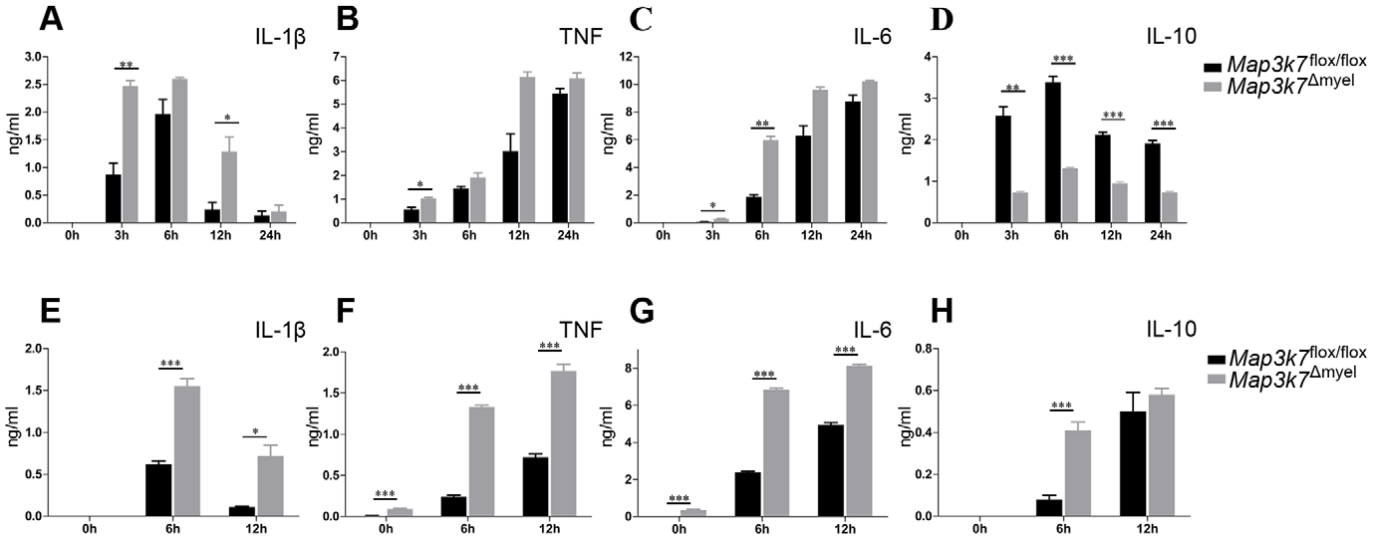
Enhanced inflammatory cytokine profile in TAK1-deficient macrophages and neutrophils in response to LPS. (A–D) BMDMs and (E–H) thioglycollate-elicited peritoneal neutrophils from *Map3k7* ^Δmyel^ and *Map3k7* ^flox/flox^ mice were stimulated with 100 ng/ml LPS. (A, E) IL-1β, (B, F) TNF, (C, G) IL-6, and (D, H) IL-10 production were measured in cell culture supernatants by ELISA. (A–H) Data are representative of three independent experiments with 4 mice per group and are shown as mean ± SEM. ^*^, p≤0.05; ^**^, p≤0.01; ^***^, p≤0.005.

We focused our subsequent analyses on identifying the role of TAK1 in LPS-induced inflammatory responses. For these studies we prepared BMDMs from *Map3k7* ^Δmyel^ and *Map3k7* ^flox/flox^ littermate mice and treated them with 100 ng/ml LPS. *Map3k7* ^Δmyel^ BMDMs exhibited reduced NF-κB (Fig. 2A) and JNK activation (Fig. 2B), consistent with similar studies in mouse monocytic cell lines for JNK activation [15], but also in other cell types such as B cells [8] and MEFs [11]. Next, we performed a detailed kinetic analysis of cytokine production upon LPS stimulation. TAK1 deficiency resulted in increased ΙL-1β, TNF, and IL-6, and decreased IL-10 production following LPS stimulation at different time points (Fig. 3A, B, C, D). A substantial increase in IL-1β production was evident at all time points up to 12 h post stimulation in *Map3k7* ^Δmyel^ BMDMs compared to control cells, whereas at 24 h, IL-1β declined to almost undetectable levels in both *Map3k7* ^Δmyel^ and *Map3k7* ^flox/flox^ cells (Fig. 3A). Increased production of TNF and IL-6 was also observed at early time points after LPS stimulation in *Map3k7* ^Δmyel^ BMDMs (Fig. 3B, C). In contrast, IL-10 production was almost 4-fold lower 3 h post LPS stimulation in *Map3k7* ^Δmyel^ BMDMs compared to control cells, and a significant reduction was still evident until 24 h (Fig. 3D). Thus upon LPS stimulation, *Map3k7* ^Δmyel^ BMDMs exhibit a hyperinflammatory phenotype.

It was previously shown that upon LPS stimulation, reduced NF-κB activation in IKKβ-deficient macrophages leads to increased IL-1β production due to enhanced IL-1β processing [23]. Therefore, reduced NF-κB activation in TAK1-deficient macrophages could at least partly account for the increased IL-1β production observed. Interestingly, a recent study revealed a regulatory loop for the induction of IL-10 during the LPS response which involves TAK1 and AUF1 [24]. Following LPS stimulation, the RNA-binding protein AUF1 maintains proper levels of TAK1 by post-transcriptional control on *Tak1* mRNA and in this way accomplishes proper NF-κB activation required for the induction of IL-10. Thus, in the absence of TAK1 several layers of transcriptional and/or post-transcriptional controls may contribute to the observed deregulated cytokine profile.

As TAK1 is deleted in both macrophages and neutrophils in *Map3k7* ^Δmyel^ mice, we next sought to determine whether TAK1 deficiency caused a similar cytokine profile change in neutrophils as in macrophages. For this we used thioglycollate-elicited peritoneal neutrophils from *Map3k7* ^Δmyel^ and *Map3k7* ^flox/flox^ mice and measured cytokine production 6 h and 12 h upon LPS stimulation. Paralleling the results of macrophages, LPS induced enhanced production of the proinflammatory cytokines IL-1β, TNF and IL-6 in *Map3k7* ^Δmyel^ peritoneal neutrophils (Fig. 3E, F, G). Notably however no reduction in the levels of IL-10 was observed in peritoneal neutrophils. In contrast, IL-10 production was significantly increased by 6 h in *Map3k7* ^Δmyel^ compared to *Map3k7* ^flox/flox^ neutrophils (Fig. 3H), suggesting that different mechanisms govern cytokine production in LPS-stimulated macrophages and neutrophils as it has been already reported for IL-1β production [23]. Interestingly, *Map3k7* ^Δmyel^ peritoneal neutrophils exhibited higher basal levels of TNF and IL-6 compared to almost undetectable levels in *Map3k7* ^flox/flox^ neutrophils. Bearing in mind that thioglycollate-elicited neutrophils are not in a steady state, this could imply a general mechanism of deregulated cytokine production upon activation of myeloid cells, in the absence of TAK1.

These results demonstrate that upon LPS stimulation, TAK1 deficiency alters the cytokine profile in macrophages and neutrophils in favor of a proinflammatory profile. This is a quite unexpected finding, as TAK1 is traditionally considered a proinflammatory molecule [8], [11]. In B cells, TAK1 deficiency was reported to result in diminished IL-6 production upon LPS stimulation [8]. Additional studies are required to delineate the molecular pathways underlying the deregulated cytokine production upon LPS stimulation in the absence of myeloid TAK1.

### Increased mortality to endotoxemia and deregulated cytokine production in mice defective in myeloid TAK1

**Figure 4:**
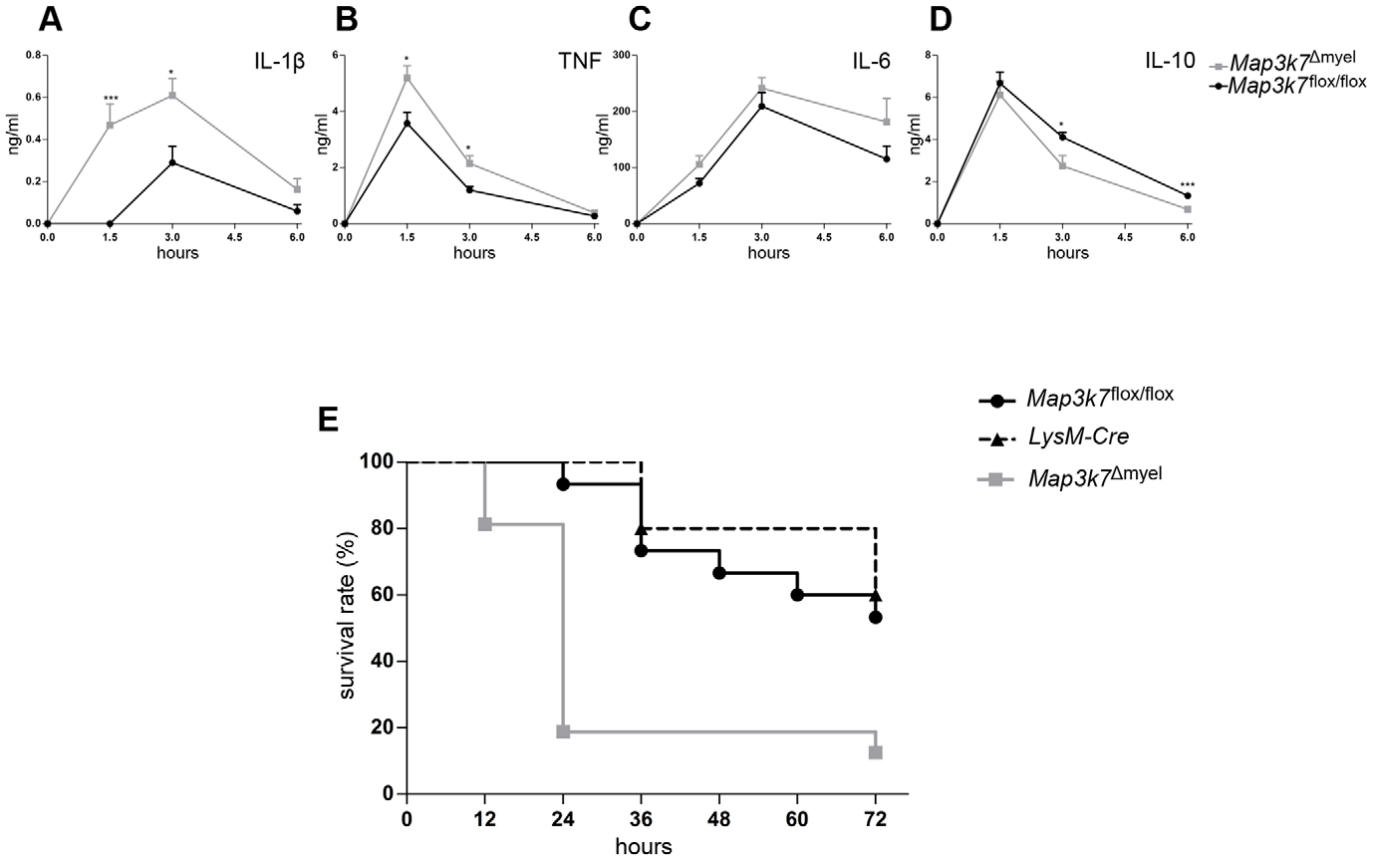
Increased LPS-induced mortality and altered circulating cytokine levels in mice deficient in myeloid TAK1. (A) IL-1β, (B) TNF, (C) IL-6, and (D) IL-10 serum levels after LPS administration (100 ìg/mouse) in *Map3k7* ^Δmyel^ and *Map3k7* ^flox/flox^ mice. Data are represented as mean ± SEM of 8–15 mice, pooled from three independent experiments. ^*^, p≤0.05; ^**^, p≤0.01; ^***^, p≤0.005. (E) Survival of *Map3k7* ^Δmyel^ (n = 16), *Map3k7* ^flox/flox^ (n = 15) and *LysM-*Cre (n = 5) mice after LPS injection (20 mg/kg).

We next sought to investigate the pathophysiological significance of the inflammatory phenotype exhibited by LPS-induced macrophages and neutrophils. For this, mice were intraperitoneally (i.p.) challenged with 100 ìg of LPS and serum samples were obtained at various intervals (0, 1.5, 3, and 6 h) after injection, for the assessment of the concentrations of circulating cytokines (Fig. 4A, B, C, D). The induction of proinflammatory cytokines was greatly enhanced in *Map3k7* ^Δmyel^ mice compared to the *Map3k7* ^flox/flox^ control mice, consistent with the *in vitro* data. A marked increase in IL-1β was observed already within the first 1.5 h, and continued to increase in the serum of *Map3k7* ^Δmyel^ mice, until it reached a peak concentration at 3 h that was 2-fold higher than the amount detected in *Map3k7* ^flox/flox^ mice (Fig. 4A). The levels of serum TNF were also significantly higher in *Map3k7* ^Δmyel^ mice compared to *Map3k7* ^flox/flox^ mice. TNF peaked at 1.5 h both in *Map3k7* ^Δmyel^ and *Map3k7* ^flox/flox^ mice, but a 1.5-fold increase was observed in the *Map3k7* ^Δmyel^ mice after which, TNF levels declined and by 6 h became identical in *Map3k7* ^Δmyel^ and *Map3k7* ^flox/flox^ mice (Fig. 4B). Additionally, a trend towards an increase in serum IL-6 was observed, although this result did not reach statistical significance (Fig. 4C). In contrast to the augmented production of proinflammatory cytokines, circulating levels of the antiinflammatory IL-10 were significantly reduced in the serum of *Map3k7* ^Δmyel^ mice compared to the *Map3k7* ^flox/flox^ controls. At 1.5 h post LPS challenge, IL-10 levels were identical but a more acute decline was observed in the serum of the *Map3k7* ^Δmyel^ mice at 3–6 h (Fig. 4D).

High circulating cytokine levels are associated with endotoxemia and contribute to the increased mortality associated with this condition. *Map3k7* ^Δmyel^ mice and *Map3k7* ^flox/flox^ littermates, as well as *LysM-*Cre control mice, were i.p. challenged with a high dose of LPS (20 mg/kg of body weight) and survival was monitored for 72 h. In this acute inflammation model, death occurs within a few days depending on the dose response to LPS. *Map3k7* ^Δmyel^ mice were indeed highly sensitive to LPS challenge, as only 19% (3 out of 16) survived within 24 h, compared to a survival rate of 93% (14 out of 15) for the *Map3k7* ^flox/flox^ mice and 100% (5 out of 5) for the *LysM-*Cre control mice (Fig. 4E). Over a period of 72 h, the survival of *Map3k7* ^Δmyel^ mice was 12%, significantly lower than the 50% survival of *Map3k7* ^flox/flox^ mice and 60% for *LysM-*Cre mice (Fig. 4E).

In conclusion, these data demonstrate that myeloid TAK1 is an essential regulator of LPS-induced inflammatory responses. Notably, an increased sensitivity to LPS-induced endotoxemia is also observed in myeloid-specific IKKβ-deficient mice [23]. Similarly, reduced NF-κB activation in the absence of TAK1 could account for the deregulated IL-1β production and the resulting increase in sensitivity to LPS challenge. Yet, myeloid-specific TAK1-deficiency also affects the production of other cytokines (TNF, IL-6) reflecting additional regulatory pathways that are simultaneously affected by TAK1.

Recently, Courties et al. reported that RNA interference-mediated knockdown of myeloid cell derived-TAK1 ameliorates inflammation and bone damage in collagen-induced arthritis [15], a finding that could raise interest in targeting TAK1 in chronic inflammatory diseases, such as rheumatoid arthritis. Further analysis revealed that knockdown of myeloid TAK1 directly attenuated Th1 responses, which play a significant role in mediating inflammation and development of collagen-induced arthritis, highlighting a modulatory role for myeloid-specific TAK1 in the adaptive immune response. Similar to our present results, in the study by Courties et al. [15] , LPS-induced JNK activation was also found to be reduced in a mouse monocytic cell line that was transiently transfected with siRNAs against TAK1. However, in the same study, despite a downregulated systemic proinflammatory response, the myeloid-specific contribution in proinflammatory cytokines was not addressed. It would be interesting to compare myeloid-specific cytokine responses in a chronic setting, such as in the collagen-induced arthritis model, with the enhanced acute myeloid response that we have observed with LPS. Our study combined with previous findings highlights a context and tissue dependence of the proinflammatory and antiinflammatory functions of TAK1 that could have implications for the future development of therapeutic concepts targeting TAK1 function in human inflammatory disease.

## Materials and Methods

### Ethics Statement

All animal experiments conformed to all current national and European legislation and were approved by the Prefecture of Attica (approval ID 1463A) and by the Institutional Animal Care and Use Committee of the Biomedical Sciences Research Center “Alexander Fleming” (approval ID 2350B).

### Generation of conditional TAK1-deficient mice

For the generation of conditional TAK1-deficient mice, a targeting vector was constructed in which a 0.6 kb fragment of *Map3k7* containing exon 2 was flanked by loxP sites. The targeting vector also contained an FRT-neo-FRT selection cassette next to the first loxP site and before exon 2 (Fig. 1A). An upstream 3.3 kb and a downstream 4.0 kb fragment were used as ‘arms’ for homology. Bruce-4 ES cells derived from C57BL/6 mice [25] were cultured, transfected and selected using standard protocols. Targeted ES cell clones were selected by Southern blotting with 5′ and 3′ probes, after digestion with SacI and BamHI respectively. The FRT-neo-FRT selection cassette was excised using flipper mice [26]. Generation of heterozygous floxed *Map3k7* mice (*Map3k7* ^flox/+^) was carried out by following standard procedures. The mice used in this study were 6–12 wks old and were maintained on a C57BL/6 genetic background. All mice were housed under specific pathogen-free conditions.

### Progenitor cell assays

Colony-forming assays were performed by plating single cell suspensions of BM (3–5×10^4^ cells/ml) in triplicate in 1 ml methylcellulose medium in 35 mm Petri dishes. Cells were incubated for 8 days in methylcellulose medium containing GM-CSF (MethoCult M3001) or incomplete methylcellulose medium (MethoCult M3231, Stem Cell Technology) supplemented with 10 ng/ml G-CSF or M-CSF (PeproTech). Colonies were scored on day 8.

### Flow cytometric analysis

Single cell suspensions were prepared from BM, blood, spleen and peritoneal cavity. Where required, red blood cells were excluded by Gey's lysis solution and debris was removed by cell strainer (70 µm, BD Falcon). After blockade of Fc-receptors with CD16/32 blocking antibody, cells were stained with antibodies conjugated with fluorochromes for 30 min on ice and washed twice before FACS analysis. For antibodies not directly conjugated to fluorochromes, staining with a secondary antibody was required for 20–30 min on ice, followed by a wash step. Data were collected by FACS Canto II and analyzed by using FACS Diva software (Becton Dickinson). For cell sorting a FACS Vantage SE II was used (Becton Dickinson). Cells were labelled using monoclonal antibodies against: CD11b (M1/70, BD Biosciences), Gr1 (RB6-8C5, e-Bioscience), F4/80 (BM8, e-Bioscience), Ly-6C (AL-21, BD Biosciences), c-Kit (2B8, e-Bioscience), Sca-1 (D7, e-Bioscience), TER-119 (TER119, e-Bioscience), B220 (RA3-6B2, e-Bioscience), CD3e (145-2C11, e-Bioscience), CD34 (RAM34, e-Bioscience), CD16/32 (93, e-Bioscience); these antibodies are conjugated with different markers, such as fluorescein isothiocyanate (FITC), phycoerythrin (PE), allophycocyanin (APC), APC-Alexa750, Alexa700, PE-Cy5.5 or biotin. Streptavidin coupled to FITC or APC (BD Biosciences) was used as a secondary antibody.

### Cell cultures

For BMDMs preparation, BM cells were cultured in complete RPMI in the presence of 20% medium conditioned by L929 mouse fibroblasts (as a source of M-CSF). On day 8, BMDMs were collected and used as indicated. To isolate neutrophils, mice were i.p. injected with 1 ml of 4% thioglycollate (DIFCO) and peritoneal neutrophils were flushed out 3–5 h later. The percentages of neutrophils in the peritoneal cell populations were similar between *Map3k7* ^Δmyel^ (83,4±4,2, n = 3) and *Map3k7* ^flox/flox^ (82,0±5,7, n = 3) littermates as analyzed by flow cytometry.

### Immunoblot analysis

Proteins were resolved by SDS PAGE and were transferred to nitrocellulose membranes by electroblot. Non-specific binding sites were blocked by incubation in 10 mM Tris-HCl pH 7.5, 150 mM NaCl, containing 0.5% Tween-20 and 5% dry milk. Membranes were blotted with antibodies against p-JNK (#46685, Cell Signaling), JNK (sc-7345, Santa Cruz) and TFIIB (sc-225, Santa Cruz) according to the manufacturer's instructions for each antibody.

### EMSA

Nuclear extracts were prepared and EMSA was performed as previously described [27]. The sequences of the oligonucleotides used for NF-κB with two tandemly positioned NF-κB binding sites (underlined) were as follows: NF-κBF (5′-ATCAGGGACTTTCCGCTGGGGACTTT-3′) and NF-κBR (5′-CGGAAAGTCCCCAGCGGAAAGTCCCT-3′).

### Endotoxemia

Mice (8–12 wks) were i.p. injected with a sublethal dose (20 mg/kg) of LPS (*Escherichia coli* 0111:B4, Sigma) and were monitored for survival. For serum cytokine measurements, mice were i.p. injected with 100 µg of LPS and at indicated time points were euthanized and blood serum was collected.

### Cytokine ELISA measurements

Serum cytokine levels and cytokines secreted from primary cells were determined by ELISA. Macrophages were plated in duplicate per mouse at 5×10^5^ cells/well in 24-well plates and were allowed to adhere for 3 h before stimulation. Neutrophils isolated from 4 mice were pooled and plated in quadruplicate at 1×10^6^ cells/well in 24-well plates. Cells were stimulated with 100 ng/ml LPS for the times indicated, followed by incubation with 1 mM ATP for 30 min for IL-1β measurements, and supernatants were taken for cytokine measurements. ELISA kits for TNF, IL-6, IL-10 (e-Bioscience) and IL-1β (BD Biosciences) were used according to manufacturer's instructions.

### Statistical analysis

Statistical comparisons were performed using unpaired Student's two tailed *t* test, with p values ≤0.05 considered statistically significant.
