# Correction: Myeloid Takl Acts as a Negative Regulator of the LPS Response and Mediates Resistance to Endotoxemia

**DOI:** 10.1371/annotation/ea1a4c80-8dfd-496a-a273-d74c1fd6e069

**Published:** 2012-03-29

**Authors:** Christina Eftychi, Niki Karagianni, Maria Alexiou, Maria Apostolaki, George Kollias

There was a formatting error in the title. The correct title is "Myeloid TAKl Acts as a Negative Regulator of the LPS Response and Mediates Resistance to Endotoxemia". The correct citation is: Eftychi C, Karagianni N, Alexiou M, Apostolaki M, Kollias G (2012) Myeloid TAKl Acts as a Negative Regulator of the LPS Response and Mediates Resistance to Endotoxemia. PLoS ONE 7(2): e31550. doi:10.1371/journal.pone.0031550

