# Correction: Myeloid Takl Acts as a Negative Regulator of the LPS Response and Mediates Resistance to Endotoxemia

**DOI:** 10.1371/annotation/74add513-eb1d-4fa1-ae2e-c3d2e7e735a0

**Published:** 2012-09-07

**Authors:** Christina Eftychi, Niki Karagianni, Maria Alexiou, Maria Apostolaki, George Kollias

There was an error in the title correction. The "1" in "TAK1" was replaced with an "L." The correct title is: "Myeloid TAK1 Acts as a Negative Regulator of the LPS Response and Mediates Resistance to Endotoxemia". The correct citation is: Eftychi C, Karagianni N, Alexiou M, Apostolaki M, Kollias G (2012) Myeloid TAK1 Acts as a Negative Regulator of the LPS Response and Mediates Resistance to Endotoxemia. PLoS ONE 7(2): e31550. doi:10.1371/journal.pone.0031550 

